# Transcriptome and physiological analyses reveal new insights into delayed incompatibility formed by interspecific grafting

**DOI:** 10.1038/s41598-023-31804-4

**Published:** 2023-03-20

**Authors:** Qiao Liu, Xiurong Wang, Yang Zhao, Feng Xiao, Yao Yang

**Affiliations:** 1 College of Forestry, Guizhou University, Guiyang, 550025 Guizhou China; 2 Institute for Forest Resources and Environment of Guizhou, Guizhou University, Guiyang, Guizhou China; 3 Key Laboratory of Forest Cultivation in Plateau Mountain of Guizhou Province, Guiyang, Guizhou China; 4 Key Laboratory of Plant Resource Conservation and Germplasm Innovation in Mountainous Region (Ministry of Education), Guizhou University, Guiyang, Guizhou China

**Keywords:** Plant sciences, Plant breeding, RNA sequencing

## Abstract

*Pinus elliottii* used as rootstock instead of homologous rootstock, have been proved to accelerate early growth of the scion (*Pinus massoniana*), for cultivation of large diameter wood. However, the basal diameter of scions in heterologous grafts was significantly smaller than self-graft 10 years later, according to field investigation, which was opposed to cultivation objectives. Although advantage of heterologous grafts has been reported, less is known about the long term effect of heterologous rootstock on scions of *P. massoniana*. The aim of present study was to investigate the mechanism of the above difference. Toward this aim, the growth traits and physiological characteristics of scions in the two graft groups were studied, and the underlying mechanism was preliminarily explored through transcriptome sequencing technology. Results showed that scions of heterologous grafts had less TSCA compared to self-grafts, while no significant difference of plant height, number of branches and canopy volume between two graft groups. Besides, scion leaves of heterologous grafts displayed higher antioxidant enzyme activity and lower chlorophyll content. And interactions between rootstocks and scions had also changed the mineral element composition of scion leaves. Compared with homologous grafts, scion leaves of heterologous grafts accumulated more K^+^, Mg^2+^ and Zn^2+^, but less Ca^2+^,which have been proved to be conducive to the growth of stem diameter of *P. massoniana*. Moreover, a comparative transcriptome analysis of two graft groups showed that DEGs between them were mainly caused by the specificity of rootstock. GO and KEGG analysis found that heterologous rootstock had different gene expression preferences, and the gene expression level between rootstocks and scions were significantly different, such as auxin auxin-related genes and stress responsive genes. That may imply that auxin pathway played an important role not only in grafting healing process, but also in maintaining the growth between scion and stock. Summary of all above results, we concluded that the long term effect of heterologous rootstock on scions may be unsatisfactory with the later rapidly growth of scion, probably due to delayed graft incompatibility between scion and stock of heterologous grafts. This study may remind us that the long-term growth of the scion deserves attention as well as the healing process, which could also provide a basis for delayed graft incompatibility.

## Introduction

Grafting is an effective and essential technique used worldwide in the planting industries to increase the yield, enhance biotic and abiotic stress resistance and modify the scion architecture^1–5^. Many studies have indicated profound influence of rootstocks on scion cultivars. Because the scion-rootstock interaction influences tree physiology, absorption capacity of mineral elements, yield efficiency, maturity and fruit quality^4,6–10^.

*Pinus massoniana* (Lamd.) is one of the most widely distributed tree species in the genus of *Pinus* in China, which plays a pivotal role in ecological environment construction and sustainable forestry production^11^. A great deal of research has been conducted on germplasm resources, genetic determination and selection, and improved varieties breeding^12^. Meanwhile, multi-generation clonal seed orchards based on homologous graft have been established^13^. Nevertheless, the slow growth of *P. massoniana* at early stages seriously hindered its popularization and application. Interspecific hybridization is difficult because of its cross-incompatibility, long cross breeding cycles, and difficult separation of progeny traits^14^. Graft is one of the most widely useful methods of asexual reproduction, aim to make genetic improvement of germplasm resources so as to improve offspring and improve their performance^15^. The key to success of grafting is the compatibility between rootstock and scion^16^. Therefore, the selection of rootstock is extremely important^17^. As a member of the double vascular subgenus,

*Pinus elliottii* (Engelm.) grows faster and has a better trunk structure than *P. massoniana*. In addition, its afforestation application areas overlap partially with that of *P.massonian*^18^. It was also reported that the 2.5-year-old grafted trees were 60.8% and 197.2% larger of diameter than the homologous graft trees, when *P. elliottii* were used to graft *P. massonian* by researchers of Guangxi Academy of Forestry^13^. Thus, the heterologous graft might be a good idea to ameliorate the characteristics of slow growth of Masson's pine at early growth stage. Although the success of heterologous grafts, we found that scions grafted on *P. elliottii* were not better than those self-grafted tress, 10 years later after graft, particularly in the trunk cross-sectional area (TSCA). It is very necessary to study the causes of this problem, because large diameter wood is the ultimate goal of commercial foresters depend on basic research.

Based on 10 years of heterologous and homologous grafted seedlings, we were able to detect the long term effect of heterologous rootstocks on scion by physiological and biochemical methods, and preliminarily explore the underlying mechanism together with transcriptome sequencing technology. And the results were supposed to have important practical application value and theoretical significance.

## Materials and methods

### Plant materials

This study has complied with relevant institutional, national, and international guidelines and legislation. The collection of materials has been approved by relevant departments. The material were collected from the gene collection area (26°16′ N, 107°31′ E) of *P. massoniana* national fine variety base in Duyun City, Guizhou Province, China, on September 2019. The scions used in the experiment were all from the same clone of Masson's pine. Thrity 10-year-old trees in heterologous grafts *P. massoniana* (scion)/*P. elliottii* (stock) and thirty 10-year-old trees in homologous grafts were selected for growth traits measurement. Mature leaves were collected from these trees for physiological measurement. Phloem tissues of 5 cm above and below the graft union of three trees in each grafted groups were collected for RNAseq. And all collected samples were quickly frozen by liquid nitrogen and stored at − 80 °C.

### Measurement of growth parameters

For estimating canopy volume, the tree height was measured from the collar region at the base to the longest shoots at top. Diameter at breast height (DBH) and canopy diameter were both measured by measuring scale in N–S and E–W directions. TCSA was measured using the following formula: TCSA = π (d/2)2, where d = average of cross measurement of trunk in N–S and E–W directions. Canopy volume (CV) was calculated according to the equation Canopy volume = 4/3*π*a^2^b, Where a: spread (E-W) + spread (N-S)/2, b: 1/2tree height.

### Chlorophyll content

The leaf chlorophyll content was estimated by 95% ethanol extraction method^19^. The extinction values of samples were measured by UV Spectrophotometer at 470 nm, 649 nm and 665 nm for pigment concentrations Chlorophyll a (C_a_) and Chlorophyll b (C_b_) respectively. And the Chlorophyll content calculation formula was below:

$${\text{C}}_{{\text{a}}} = {13}.{\text{95A}}_{{{665}}} - {6}.{\text{88A}}_{{{649}}}$$

$${\text{C}}_{{\text{b}}} = {24}.{96}\;{\text{A}}_{{{649}}} - {7}.{32}\;{\text{A}}_{{{665}}}$$

$${\text{C}}_{{{\text{Total}}\;{\text{Chl}}}} = {\text{C}}_{{\text{a}}} + {\text{C}}_{{\text{b}}}$$

$${\text{Chlorophyll}}\;{\text{content}} = {\text{C}}_{{{\text{Total}}\;{\text{Chl}}}} \times 0.0{25} \times {1}/0.{2}$$

### Leaf nutrient analysis

Leaves in each tree were randomly collected from all directions, washed with tape water and followed by double distilled water. The leaves were then dried at 105° ± 1 °C in oven for 0.5 h and then at 60 °C to constant weight.

Nitrogen content in leaves was determined by using Digestion Block method^20^.Phosphorus in leaves was estimated by vando-molybdo-phosphoric yellow color method^21^.Total K^+^ contents in leaves were estimated according to Jackson^22^. For quantification of Ca^2+^and K^+^ in leaves, 1.0 g dry weight was digested in diacid (using HNO3 and HClO4 in the ratio of 9:4). K^+^ were analysed by a micro-processor based flame photometer. And Ca^2+^, Mg^2+^, Zn^2+^, Cu^2+^and Fe^2+^contents were determined by atomic absorption spec-trophotometer.

### Biochemical parameters

#### Determination of SOD, POD, and CAT activities

In each sample, 0.5 g fresh leaf was ground with a pestle in an ice-cold mortar with 4 ml of 50 mM phosphate buffer (pH 7.0). Activations of enzymes were measured in the supernatant of homogenates centrifuged for 20 min at 4 °C at 12,000 rpm. SOD activity was assayed by measuring its ability to inhibit the photochemical reduction of nitro blue tetrazolium^23^. POD activity was measured as the increase in absorbance at 470 nm caused by guaiacol oxidation^24^. CAT activity was measured as the decline in absorbance at 240 nm caused by a decrease in H_2_O_2_ removal^25^.

#### Total soluble proteins and Total soluble sugar

Samples were placed in 1.5 mL microfuge tubes and crushed in 100 mM sodium phosphate buffer (pH 7.8) for protein and carbohydrate extraction. At 4 C, samples were centrifuged for 15 min at 16,000 rpm. The supernatants were removed and analyzed. Protein samples were diluted 1:50 and carbohydrate samples were diluted 1:25 in the sodium phosphate buffer before analysis. Protein levels were determined using a BCA assay following the manufacturer’s directions with absorbance readings at 595 nm and quantified by comparison to BCA standards^26^. Soluble sugar were measured according to Laurentin and Edwards^27^ and following the manufacturer’s directions with absorbance readings at 620 nm and quantified by comparison to glucose standards.

### Total RNA extraction and RNA library construction

#### Sample preparation

Total RNA was extracted from the above samples using the TRIZOL kit (Invitrogen) and then treated with DNaseI to remove DNA. Before preparation of the RNA libraries, total RNA samples were quantified. 1% agarose gel was used to monitor degradation and contamination of the RNA samples. The mRNA was enriched with magnetic beads of Oligo (dT) beads after the quality test. The fragmentation buffer was added to break the mRNA into short pieces. Using mRNA as template, cDNA was synthesized by reverse transcription using six base random primers. Illuminape library was established after double stranded cDNA purified, terminal repair. After passing quality inspection, Illumina PE library was constructed and 2 × 150 bp cDNA library was sequenced. The raw data generated in this study have been uploaded to Sequence Read Archive (https://www.ncbi.nlm.nih.gov/sra/). The accession number is BioProject ID: PRJNA792704.

#### Data analysis

The quality of the original sequencing data of each sample was evaluated by fastx _ toolkit_0.0.14 (http://hannonlab.cshl.edu/fastx_toolkit/); Seqprep (https://github.com/jstjohn/SeqPrep) was used for quality control on the original sequencing data, the reads with linker, N ratio greater than 0.1% and low-quality sequences were removed to obtain clean reads. Bowtie2 (https://sourceforge.net/projects/bowtie-bio/files/bowtie2/2.3.5.1/) was used to compare the full-length transcriptome of the third generation of *P*.*massoniana* obtained for mapping and RSEM for transcription. In this quantity, the obtained read Count value is FPKM to analyze gene expression levels. The gene differential expression analysis of multiple samples (≥ 2) was carried out by DESeq2 (http://bioconductor.org/packages/stats/bioc/DESeq2/) , which was defined as differentially expressed genes (DEGs) according to the screening criteria of|log2.Fold_change|> 1 and q.value < 0.005. Differential expression gene (DEGs) were assigned to gene ontology (GO) enrichment analysis using the GOseq R package, and GO terms with corrected *P*-values < 0.05 were considered significantly enriched. The statistical enrichment of DEGs in

KEGG pathways (http://www.genome.jp/kegg/) was tested using KOBAS software.

## Data analysis

All data were analyzed with SPSS Statistical Software (Version24.0). Student’s t-test was used to compare means between two graft groups. Data visualization and GSEA analysis were performed using the Majorbio Cloud Platform (https://cloud.majorbio.com ) and GraphPad Prism 8 (https://www.graphpad.com/scientific-software/prism/).

## Results

### Morphological characteristic changes of scion between two grafted groups

Less TSCA of scions was found in heterologous grafts compared to homologous graft (Table 1). Plant height, number of branches, and canopy volume did not differ significantly between two graft groups. It showed that the long-term impact of heterogenous rootstock on scions was mainly reflected in the transverse growth of scions.

**Table 1:** The phenotype of scions in two grafted groups 10 years after graft.

Grafted group	Plant hight (m)	Total branches number (N)	Canopy volume (m^3^)	TCSA (cm^2^)
Homologous graft	6.02 ± 0.24	7.00 ± 1.00	252.42 ± 71.63	125.12 ± 5.21^a^
Heterologous graft	5.76 ± 0.70	7.60 ± 0.57	271.52 ± 117.93	104.55 ± 6.97^b^

A significant difference between graft groups was indicated by different lowercase letters (*P* < 0.05) using the Student’ s t-test, no letter represent no difference. Values are means ± SE.

### Photosynthetic pigments concentration of scion leaves

**Figure 1:**
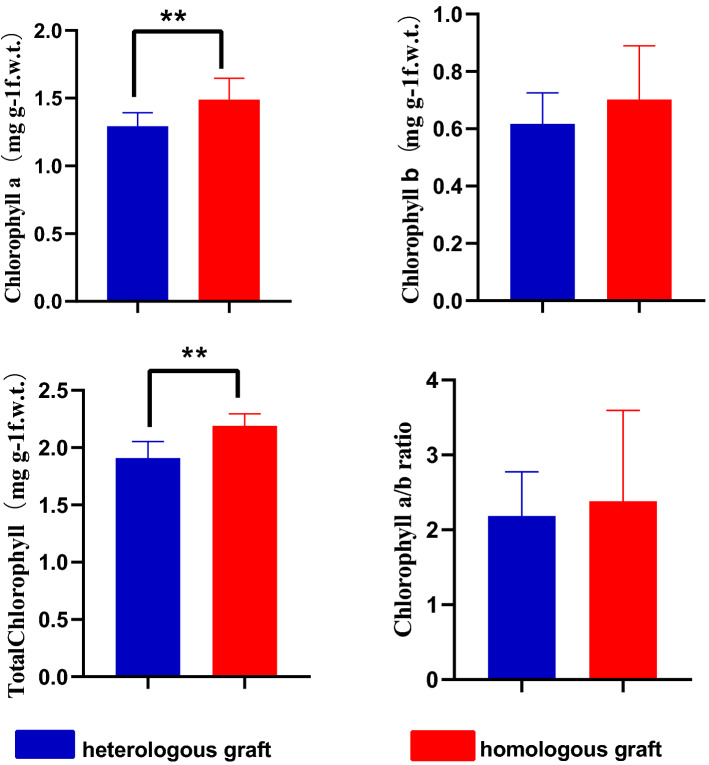
Chlorophyll a(Chl a),Chlorophyll b(Chl b),Total chlorophyll (T Chl) and chlorophyll a/b ratio (Chla/b) of scion leaves in two grafted groups. A significant difference between grafted groups was indicated by ** (*P* < 0.01) using the Student’ s t-test.

Lower chlorophyll a and total chlorophyll content were both found in scion leaves of heterologous grafts compared to homologous grafts (Fig. 1). However, chlorophyll b, chlorophyll a/b ratio had no significantly difference between two graft groups. Higher photosynthetic pigment content may mean more photosynthetic products under the same conditions, which may be more conducive to the lateral growth of plants when maintaining almost the same other growth traits.

### Scion leaf nutrient analysis

**Figure 2:**
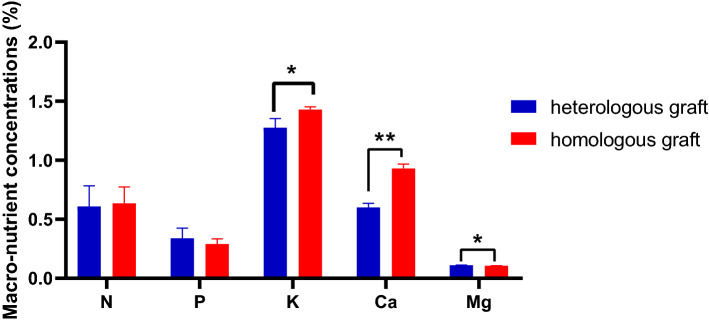
Rootstock influence on leaf macro-nutrient concentrations of scions. A significant difference between grafted groups was indicated by* (*P* < 0.05) or ** (*P* < 0.01) using the Student’ s t-test.

**Figure 3:**
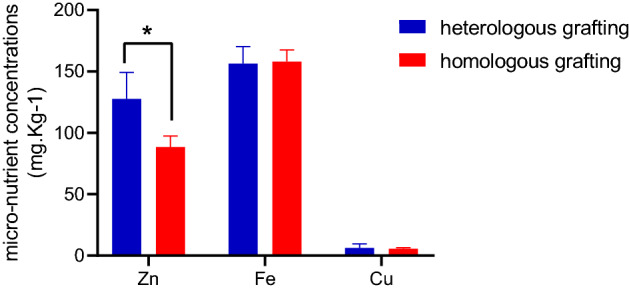
Rootstock influence on leaf micro-nutrient concentrations of scions A significant difference between grafted groups was indicated by*(*P* < 0.05) using the Student’ s t-test.

Scion leaves were found to be similar statistically in terms of total N and total P content, indicating that these two elements were little influenced by rootstock. However, scion leaves of heterologous grafts significantly accumulated more Mg^2+^, but lower K^+^ and Ca^2+^ when compared to homologous grafts (Fig. 2). In addition, the scion leaves micro-nutrients were also affected by heterologous rootstock (Fig. 3).Both Cu^2+^ and Fe^3+^ were not significantly different, while more Zn^2+^ accumulation in scion leaves of heterologous grafts than homologous grafts.

Thus, the content of different ions in scion leaves differed between two graft groups due to rootstocks, because different rootstocks had different absorption preferences for ions.

### Biochemical parameters

**Figure 4:**
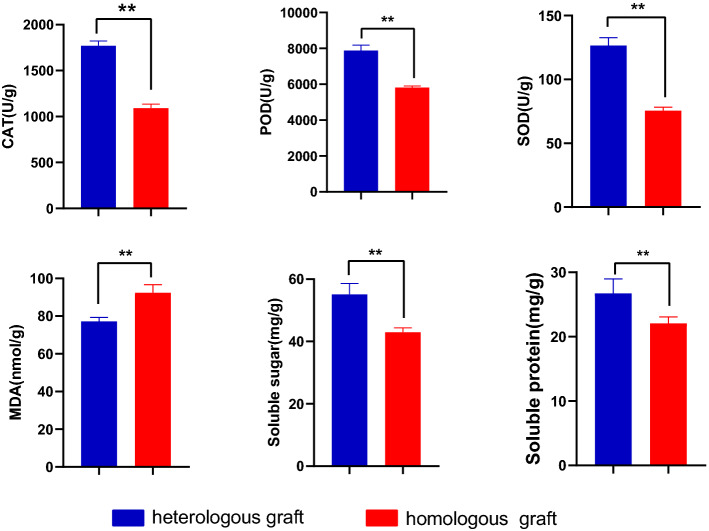
Catalase (CAT) activity, Superoxide dismutase (SOD) activity, Polyphenoloxidase (POD) activity, MDA, total soluble sugar and protein content in the scion leaves of two graft groups. A significant difference between grafted groups was indicated by ** (*P* < 0.01) using the Student’ s t-test.

Much higher superoxide dismutase (SOD), polyphenoloxidase (POD), and catalase(CAT) activity and lower malondialdehyde (MDA) content of scion leaves in heterologous grafts were found than those in homologous grafts (Fig. 4), indicating that higher resistance of against oxidative stress had been induced by heterologous rootstocks. Additionally, scion leaves of total soluble sugar and protein content were also more than leaves of homologous grafts, which indicated that these substances were easily affected by heterologous rootstocks. Because antioxidant enzyme systems and soluble substances were often associated with stress resistance, we could speculate that heterologous rootstocks improved the stress resistance of scions compared to homologous rootstocks in the same living environment. And the increase in resistance could be maintained for many years after grafting.

### Correlation between measurement indexes

**Figure 5:**
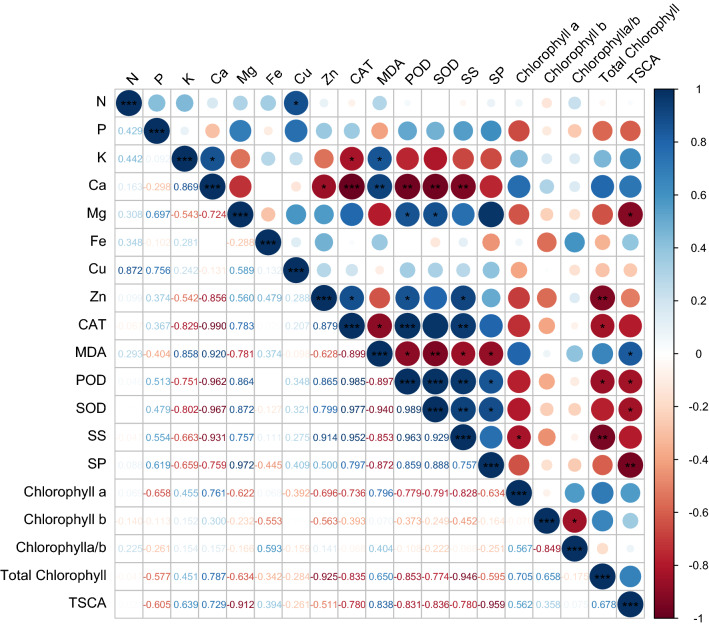
Correlation analysis heat map, SS and SP represent for soluble sugar and soluble protein.

Aim to investigate the correlativity between scion growth traits and these physiological and biochemical indexes, a correlation analysis was conducted (Fig. 5). The result showed that antioxidant enzymes exhibited strong positive correlations with one another, whereas strong negative correlations with TSCA. That was to say that higher antioxidant enzymes activity of leaves may not be conducive to the lateral growth of the stem. TSCA had strongly negative correlation with Mg and soluble protein, but positive correlation with MDA. MDA showed positive correlation with K and Ca. Ca showed positive correlation with K, but negative correlation with Zn. These results indicated that proper MDA content could be good for stem diameter by affecting absorption of mineral elements. Total chlorophyll correlated positively with TSCA, but negatively with Zn, soluble sugar and antioxidant enzymes, which meant that antioxidant enzymes and soluble sugars may indirectly regulate photosynthesis by affecting the content of photosynthetic pigments to play an important role in TSCA. To put it simply, there was a complex network among mineral elements accumulation, photosynthetic pigment, soluble sugars and proteins as well as antioxidant enzymes activity to regulate the growth of scion. Heterologous rootstock had affected the synthesis and metabolism of scion, result in different growth trait.

### Illumina sequencing, mapping and quantitative assessment of transcriptomes

**Figure 6:**
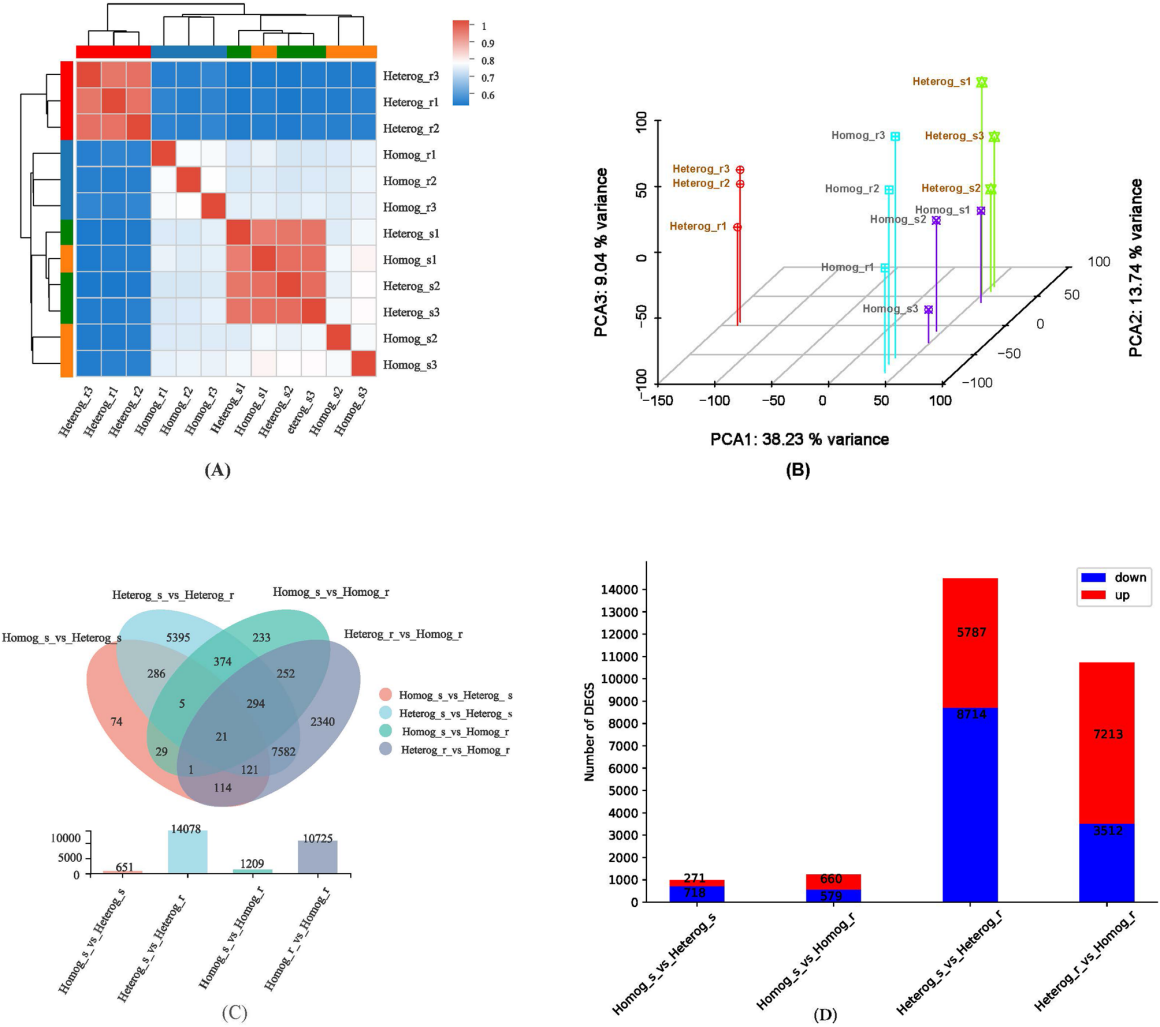
(**A**) Correlation of samples expression; (**B**) Principal component analysis between samples. (**C**) Number of up/down differential genes in different groups (**D**): Venen map of differentially expressed genes between different combinations.

RNA-Seq results were summarized in Table 2. A total of 44 million raw reads were generated per sample. After quality control, 6.23 to 8.77 Gb clean bases with a Q30 ratio over 89.97% and a GC content over 44.67% were obtained from each of the 12 libraries. Based on these results, the sequencing data had a high degree of coverage in the genome, and the data were accurate and sufficient for further bioinformatics analysis. The heat map analysis of correlation between samples showed that the correlation between Heterog_r group and other groups was low, which was also caused by the rootstock species specificity (Fig. 6A). According to PCA analysis (Fig. 6B), the scions grafted on heterologous rootstocks had greater intra-group differences than homologous grafts. DegSeq2 was used to screen the differentially expressed genes and the number of differentially expressed genes was counted (Fig. 6C). Among them, there were 989 DEGs between Heterog_s and Homog_s, with 271 up-regulated and 718 down-regulated genes. 1239 DEGs with 660 up-regulated genes and 579 down-regulated genes were found between Homog_r and Homog_s. 14,501 DEGs between Heterog_r and Heterog_s were found, with 5787 up-regulated genes and 8714 down-regulated genes. The number of DEGs between Heterog_r and Homog_r was 10,725, with 7213 up-regulated genes and 3512 down-regulated genes. The venendiagram of DEGs (Fig. 6D) of the four groups above was carried out. All above results indicated that more different genes between scion and rootstock were found in heterologous grafts, and more genes expression had been effected by heterologous rootstock, which then finally affected the growth of scion.

**Table 2:** Sample libraries sequencing data statistics.

Sample name	Clean reads	Error rate (%)	Q20 (%)	Q30 (%)	GC (%)	Total Mapped (%)
Heterog_r1	45968896	0.0297	96.29	89.97	44.78	57.71
Heterog_r2	48728934	0.027	97.33	92.21	45.23	63.08
Heterog_r3	50486810	0.0268	97.40	92.38	44.85	60.81
Heterog_s1	48436968	0.0267	97.43	92.42	44.92	73.55
Heterog_s2	45824480	0.0269	97.83	92.31	44.81	72.00
Heterog_s3	46773304	0.0265	97.50	92.59	45.03	73.42
Homog_r1	46909660	0.0272	97.22	91.97	44.83	72.82
Homog_r2	47992394	0.0272	97.25	92.02	44.91	74.23
Homog_r3	47074190	0.0267	97.45	92.48	45.15	74.84
Homog_s1	46458960	0.0269	97.35	92.29	44.80	72.36
Homog_s2	54099372	0.0272	97.23	92.00	44.85	72.87
Homog_s3	49381228	0.0266	97.45	92.51	44.67	71.56

### Functional annotations of DEGs

#### GO and GSEA enrichment analysis

**Figure 7:**
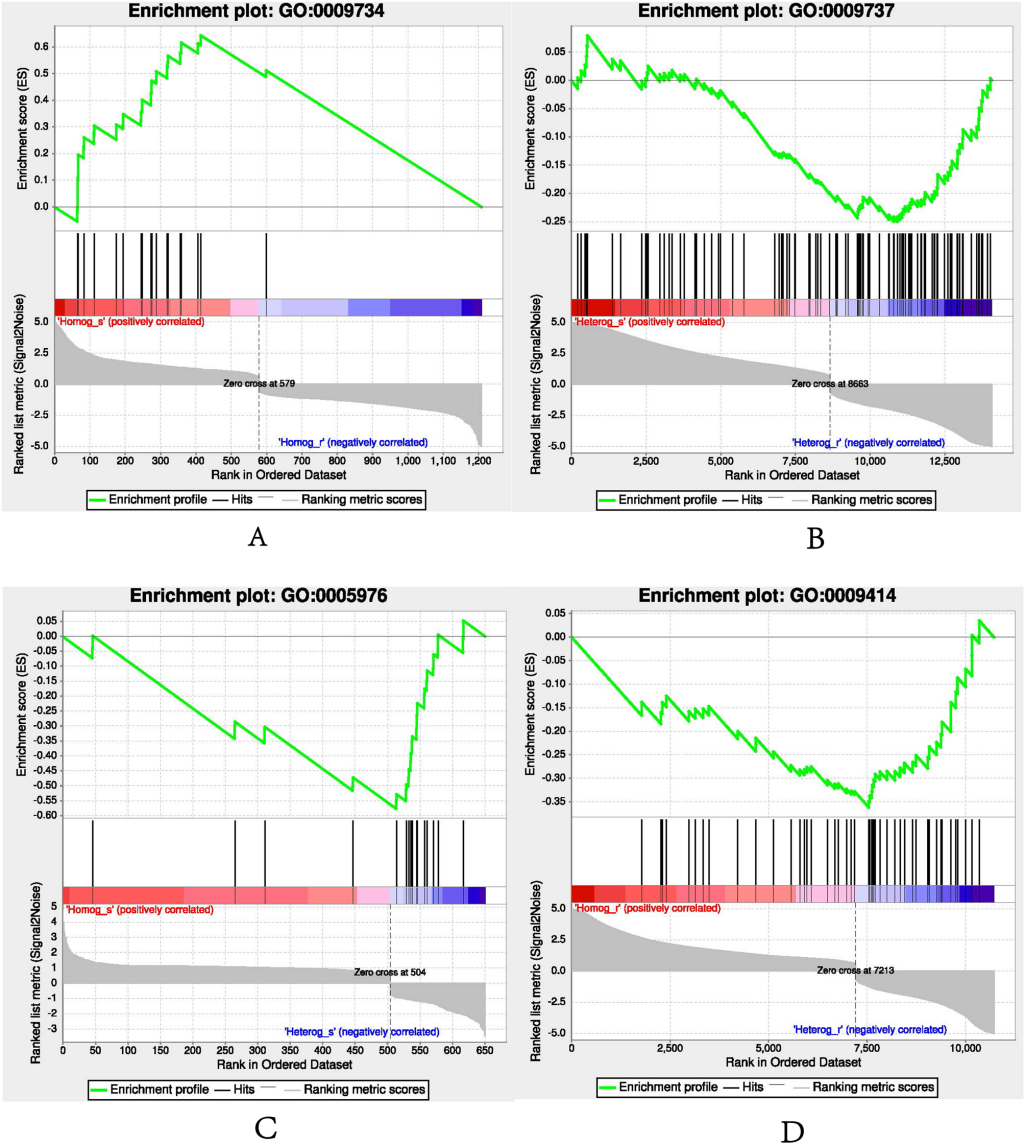
GSEA analysis of different grafting groups, (**A**) auxin-activated signaling pathway (GO:0009734) between Homog_s and Homog_r, (**B**) response to abscisic acid (GO:0009737) between Heterg_s and Heterg_r, (**C**) polysaccharide metabolic process (GO:0005976) between Homog_s and Heterg_s, (**D**) response to water deprivation (GO:0009414) between Homog_r and Heterg_r.

To evaluate gene function of these DEGs, DEGs between grafted scions and rootstocks were annotated using GO and GSEA method (Fig. 7A–D). And DEGs between Homog_s and Homog_r significantly enriched in auxin-activated signaling pathway (GO:0009734) were upregulated in Homog_s. While GSEA enrichment analysis of DEGs between Heterg_s vs Heterg_r showed that biological processes such as response to abscisic acid (GO:0009737) were upregulated in Heterg_r. DEGS between Homog_s and Heterg_s, Homog_r and Heterg_r were also analyzed. And results showed biological processes of polysaccharide metabolic process (GO:0005976), cofactor biosynthetic process (GO:0051188) and sulfur compound metabolic process (GO:0006790) were both upregulated in Heterg_s between Homog_s and Heterg_s, which may indicate more metabolic pathways have changed in Heterg_s caused by heterologous grafting. Then DEGS of rootstocks in DNA-templated transcription initiation (GO:0006352),positive regulation of nucleobase-containing compound (GO:0045935) and other molecular functions were upregulated in Homog_r, but cellular amino acid catabolic process (GO:0009063), response to water deprivation (GO:0009414) and other biological processes were upregulated in Heterg_r. All above indicated different rootstocks had different gene expression preferences and interactions between rootstocks and scions may be more complex than we imagine.

#### KEGG enrichment analysis between rootstock and scion of two grafted groups

**Figure 8:**
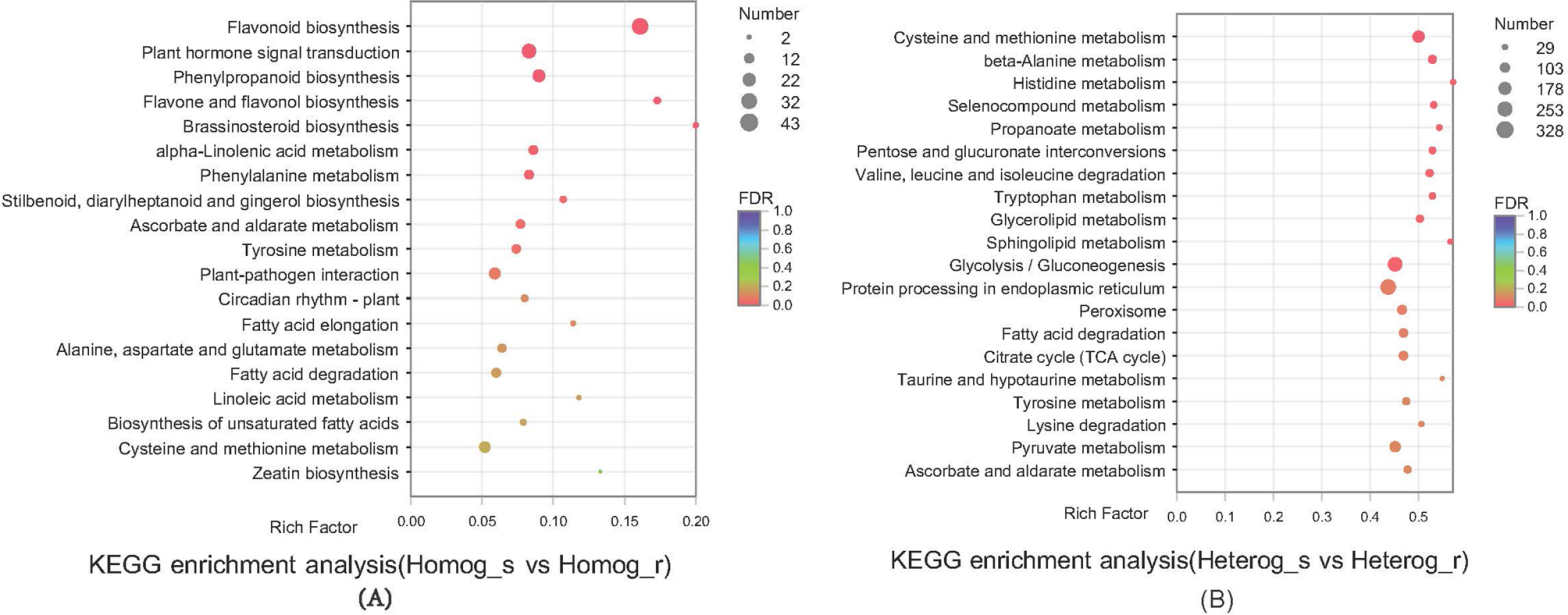
KEGG analysis of DEGs between scion and rootstock of two grafted groups, (**A**) Scion and rootstock of homologous grafts, (**B**) Scion and rootstock of heterologous grafts.

KEGG pathway enrichment analysis was employed to further understand the main biochemical metabolic and signal transduction pathways between two grafted groups (Fig. 8A,B). Ten pathways significantly enriched in upregulated DEGs between Homog_s and Homog_r, with most DEGs being involved in Flavonoid biosynthesis, Plant hormone signal transduction, Phenylpropanoid biosynthesis, Flavone and flavonol biosynthesis, Brassinosteroid biosynthesis, alpha-Linolenic acid metabolism, Phenylalanine metabolism, Stilbenoid, diarylheptanoid and gingerol biosynthesis, Ascorbate and aldarate metabolism and Tyrosine metabolism. While eleven pathways significantly enriched in upregulated DEGs between Heterg_s and Heterg_r, with most DEGs being involved in Cysteine and methionine metabolism, beta-Alanine metabolism, Histidine metabolism, Selenocompound metabolism, Propanoate metabolism, Pentose and glucuronate interconversions, Valine, leucine and isoleucine degradation, Tryptophan metabolism, Glycerolipid metabolism, Sphingolipid metabolism and Glycolysis/Gluconeogenesis.

#### Go and KEGG enrichment analysis of DEGs caused by graft

**Figure 9:**
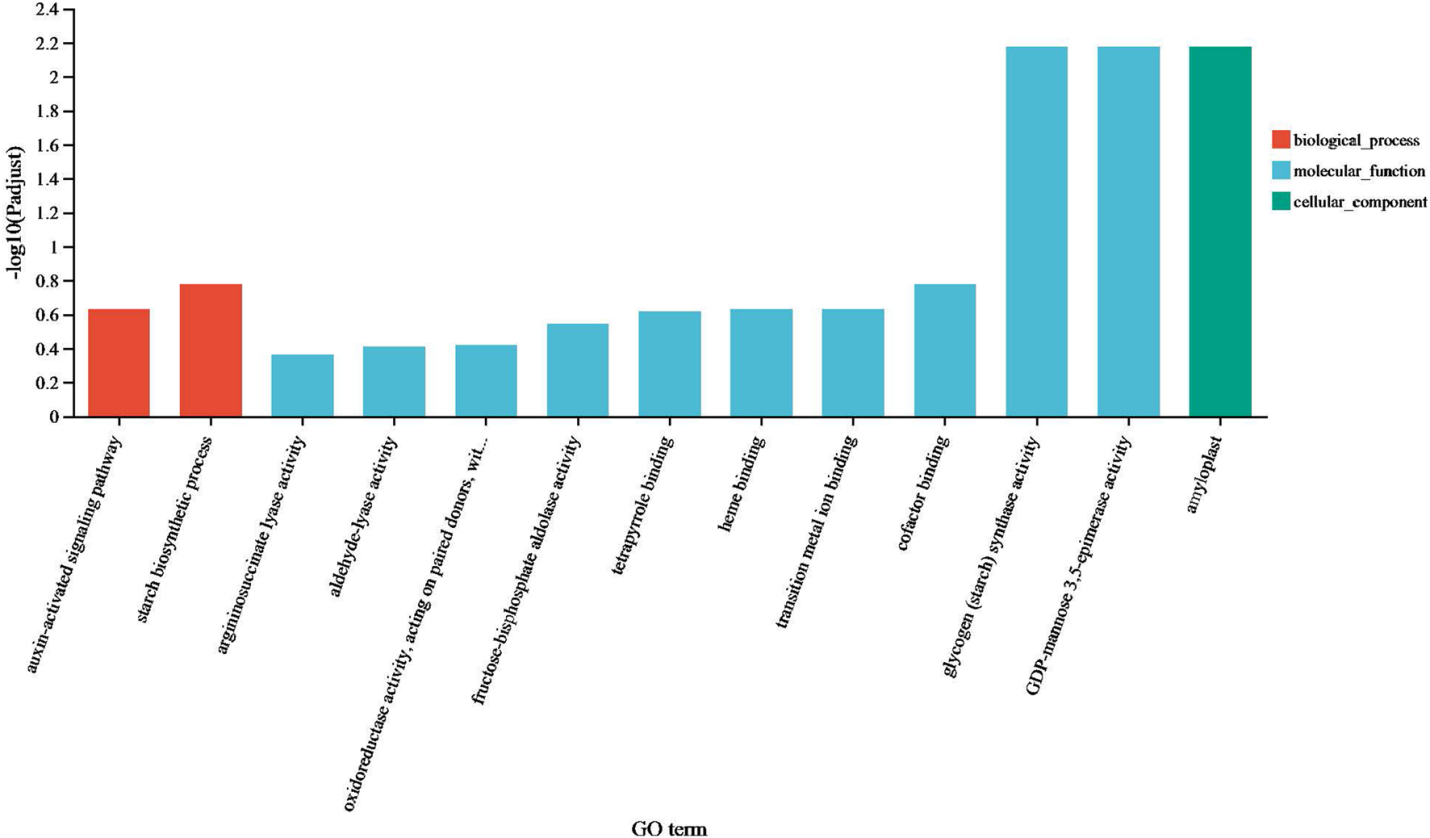
Go analysis of intersection of DEGs between rootstocks and scions of two grafted groups.

**Figure 10:**
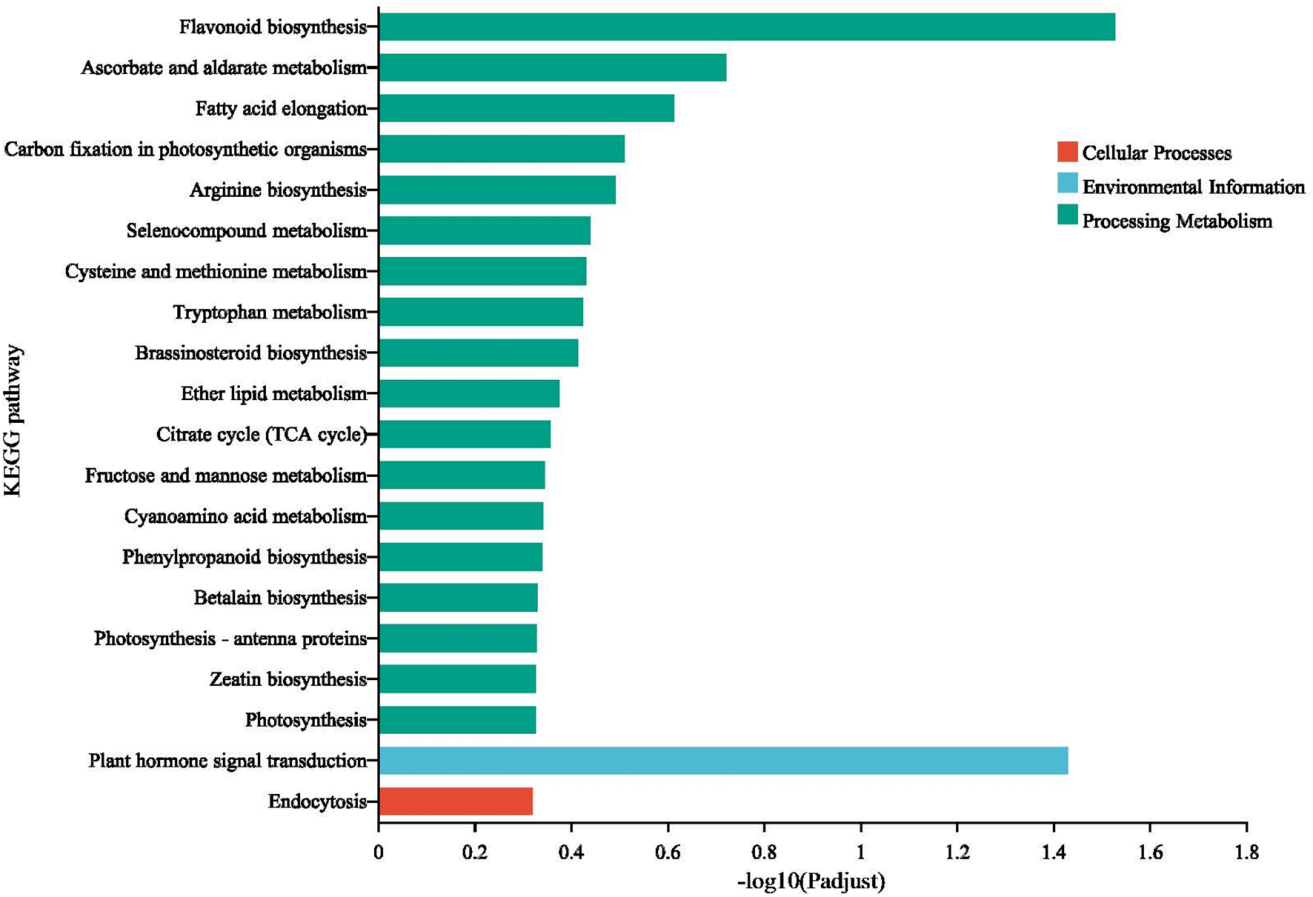
KEGG analysis of intersection of DEGs between rootstocks and scions of two grafted groups.

Due to the interaction between rootstock and scion, intersection of DEGs between Heterg_r and Heterg_s and DEGs between Homog_r and Homog_s, namely common DEGs caused by heterologous and homologous grafts, were also analyzed by GO (Fig. 9) and KEGG (Fig. 10). The results indicated that two grafts both affect genes expression between scion and rootstock, such as glycogen (starch) synthase activity, auxin-activated signaling pathway, amyloplast and so on. Flavonoid biosynthesis, Plant hormone signal transduction and Endocytosis pathways were significantly enriched by KEGG.

#### DEGs involved in Auxin signal transduction

**Figure 11:**
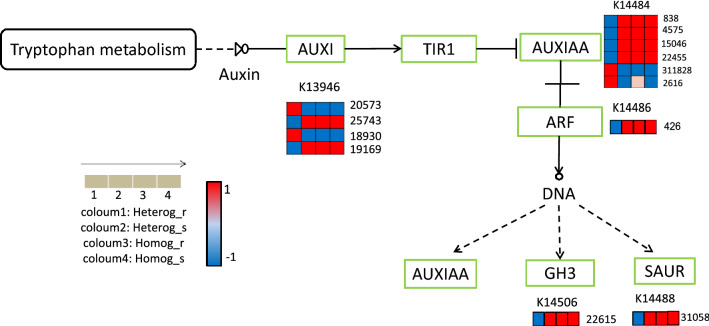
Different genes expressed in auxin related pathway.

Since auxin plays an important role during grafting reconnection, we wanted to investigate whether this hormone also affected the growth between scion and rootstock at a later stage. Thus, the auxin signal transduction pathway of plant hormones was primarily studied (Fig. 11). It was easily found that Heterg_s and Heterg_r did not exhibit consistent upregulation or downregulation of auxin-related genes compared to self-graft, which was mainly caused by reverse gene expression of Heterg_r. The same gene expression pattern between Heterg_s and Homog_s showed that Heterg_r had little effect on the auxin related genes expression of Heterg_s. But the different gene expression pattern of Heterg_r may be affected by scion, for the complex interaction between rootstock and scion.

## Discussion

In modern agriculture, grafting is widely used to improve biotic and abiotic stress tolerance, modify plant architecture, induce precocious flowering and rejuvenate old perennial varieties, boost yield, and more^28,29^. It is critical to the tree growth that the rootstock-graft interactions are compatible, including the root characteristics^4,6,30^, absorption and translocation of water and nutrients^5,31,32^ and different signals passing through the graft union^1,4^. In the present study, we measured the growth and development traits, physiology and biochemistry of *P. massoniana* scion as well as transcriptomic difference to study the long-term effect of heterologous rootstock on scion growth. Unexpectedly our results showed that the *P. elliottii* rootstock had no significantly positive effect on the development and physiology of the *P. massoniana* scion compared to homologous graft 10 years after graft. Although heterologous grafting scion had more content of soluble sugar, soluble protein and higher antioxidant enzymes activity, their TCSA were lower than homologous grafted scion, which was not good for timber forest cultivation.

Rootstock had a meaningful impact on nutrient absorption and the nutrient uptake efficiency varied with the rootstocks^28,32^. In heterologous graft groups rootstocks changed the content of mineral elements in leaves, with lower content of K^+^ and Ca^2+^ and higher content of Zn^2+^ and Mg^2+^compared to homologous graft groups, which was consistent with the previous researches that nutrition efficiency could be influenced by the rootstock^33,34^. Difference in leaf nutrient concentration of different rootstocks might be due to alteration of root morphology and hydraulic conductance. Because it have been reported that rootstocks had differential capacity of hydraulic conductance, which was positively correlated with nutrient accumulation^6,34^. As for *P. massoniana*, leaf calcium was positively correlated with thoracic diameter, but leaf zinc was inversely correlated with diameter^31^, which was consistent with our correlation analysis.

More researches showed complex physiological metabolites were affected during graft union formation^5,7,29^. Grafting, as a wounding stress, triggers antioxidant defense systems^4^, resulting a higher level of reactive oxygen species (ROS) or a less efficient detoxification system on incompatible scion/rootstock interfaces^35^. Initial healing of the graft union does not ensure long-term compatibility, because some stock/scion combinations incompatibility may appear only after several years^35,36^. In this article, higher antioxidant enzymes activity, soluble protein were found in heterologous grafting scion leaves, which could indicate that incompatibility may exist between rootstock and scion in *P. massoniana/P. elliottii* heterologous graft system due to a belated response to the auxin and carbohydrate imbalance^37,38^ caused by phloem graft union irregularities. Meanwhile, the study also revealed that rootstock-scion interactions persist throughout the composite plant's lifespan, even if graft compatibility was satisfactory^38^.

It has also been reported that grafting induces differences in the transcriptome profile of grafted parents in plants, such as grapevines^39,40^. However, much attention had been absorbed on the connection of the graft union. Until recently, little was known about the long-term changes between rootstocks and scions. RNA-Seq was used in this study to identify DEGs in heterologous and homologous grafted tissues. Functional annotation and enrichment analysis of DEGs revealed that the overrepresented genes were involved in physiological metabolism (flavonoid biosynthetic process, auxin-activated signaling pathway, cellular response to auxin stimulation) between scion and rootstock in homologous graft group, while response to water deprivation and abscisic acid between scion and rootstock in heterologous graft group. These transcriptomic results suggested that stress about water existed between scion and rootstock in heterologous grafting group due to delayed graft incompatibility, with the later rapidly growth of scion^41^. And this could be partly proved by the higher antioxidant enzymes activity, soluble protein and soluble sugar in leaves.

Then we focused on the auxin-activated signaling pathway in homologous graft. Results showed that AUX1、IAA13、IAA9 genes, bZIP transcription factor were differentially expressed between scion and rootstock in homologous graft group when compared to heterologous graft. All those auxin related genes may contribute to maintain the local auxin concentration above and below the graft port^42,43^ and regulate the normal function of nutrients and water transportation, resulting in more stem growth. Thus, IAA may also play an important role in maintaining the function of grafted plant, not only at the beginning of graft union reconnection. When compared with Heterg_s and Homog_r, water deprivation and abscisic acid response were both upregulated in Heterg_r. Together with physiological data, we could conclude that *P. elliottii* as rootstock to graft *P. massoniana* was more sensitive to water deprivation than *P. massoniana*. The observed reactions (higher antioxidant enzyme activities, soluble sugars, and protein) in scions were mainly due to upregulation of enriched genes between rootstock and scion in Heterologous graft.

## Conclusion

Complex interactions between rootstock and scion have changed mineral element composition and antioxidant enzyme activity in scion as well as transcription changes. Based on the results of compared transcriptome analysis, there may be late incompatibility between rootstock and scion of Heterologous graft. The delayed incompatibility was mainly caused by auxin imbalance between rootstocks and scions and large moisture requirement for rapid growth of scion in later period.

## Data Availability

The RNA sequence generated in this study have been uploaded to Sequence Read Archive (https://www.ncbi.nlm.nih.gov/sra/).The accession number is BioProject ID: PRJNA792704.
